# Triage of acute medical patients - interobserver Reliability

**DOI:** 10.1186/1757-7241-23-S1-A13

**Published:** 2015-07-16

**Authors:** Jacob B Brodersen, Peter Hallas, Mikkel Brabrand

**Affiliations:** 1 Department of Medical Gastroenterology, Odense University Hospital, Odense C, Denmark; 2 Department of Emergency Medicine, Sydvestjysk Sygehus, Esbjerg, Denmark; 3 Copenhagen University Hospital, Department of Pediatric Anesthesia, Rigshospitalet, Copenhagen, Denmark; 4 Department of Emergency Medicine, OUH Odense University Hospital, Odense, Denmark

## Background

In most emergency departments (ED) around the world, patients are initially assessed using a triage system or risk stratification tools. The result of the scores is an important factor in determining the course of treatment for critically ill patients. Several different systems are used, but most include blood pressure, respiratory rate, pulse, and level of consciousness and the individual systems then have a number of different parameters. Considering the vital role the results of these scores have on the course of treatment, the number of studies on inter-observer reliability is limited. The aim of this study was to measure the inter-observer variability when assessing patients using the Danish Emergency Process Triage (DEPT) (using only vital signs), the Modified Early Warning Score (MEWS), the HOTEL score, the Simple Clinical Score (SCS) and PARIS score.

## Methods

This is a questionnaire study, based on video recordings made at the admission of acutely ill medical patients to the emergency department. The videos were recorded during the first 10-15 minutes after arrival of the patient to the department of emergency medicine at Sydvestjysk Sygehus Esbjerg. The video were edited and shown in an internet based questionnaire.

Inclusion of observers started with an initial open invitation to participate in a questionnaire study, and the responders was included in the actual study. Cases were assigned at random, and each nurse used all five systems to evaluate the same patient. The Intra Class Coefficient (ICC) was calculated for each system.

## Results

Five cases were used and eight observers evaluated each case. All observers were trained nurses with a median experience of 14.65 (range 2-35) years. The individual ICC of DEPT was 0.64 (95% confidence interval (CI): 0.33-0.94), MEWS 0.00 (95% CI: 0.00-0.46), HOTEL 0.70 (95% CI: 0.39-0.95), SCS 0.08 (95% CI: 0.00-0.63) and PARIS 0.39 (95% CI: 0.11-0.87).

## Conclusions

HOTEL and DEPT - which is the triage system most widely used in Denmark - had the highest ICC of the five systems. In particular, MEWS had a poor ICC.

